# Genomic Mosaicism in Fowl Adenovirus 3 Strains

**DOI:** 10.3390/ani15040508

**Published:** 2025-02-11

**Authors:** Zalán Homonnay, Szilvia Jakab, Szilvia Marton, Marianna Domán, Krisztina Bali, Eszter Kaszab, Gábor Kemenesi, Tamás Mató, István Kiss, Vilmos Palya, Krisztián Bányai

**Affiliations:** 1 Ceva-Phylaxia Ltd., Szállás u. 5, H-1107 Budapest, Hungary; zalan.homonnay@ceva.com (Z.H.); tamas.mato@ceva.com (T.M.); istvan.kiss@ceva.com (I.K.); vilmos.palya@ceva.com (V.P.); 2 HUN-REN Veterinary Medical Research Institute, Hungária krt. 21, H-1143 Budapest, Hungary; jakabszilvia.home@gmail.com (S.J.); martonsil@gmail.com (S.M.); krisz.bali@gmail.com (K.B.); eszter.kaszab@gmail.com (E.K.); 3 National Laboratory for Infectious Animal Diseases, Antimicrobial Resistance, Veterinary Public Health and Food Chain Safety, Hungária krt. 21, H-1143 Budapest, Hungary; 4 Department of Microbiology and Infectious Diseases, University of Veterinary Medicine, Hungária krt. 23–25, H-1143 Budapest, Hungary; 5 One Health Institute, Faculty of Health Sciences, University of Debrecen, Nagyerdei krt. 98, H-4032 Debrecen, Hungary; 6 National Laboratory of Virology, Szentágothai Research Centre, University of Pécs, H-7624 Pécs, Hungary; kemenesi.gabor@gmail.com; 7 Institute of Biology, Faculty of Sciences, University of Pécs, H-7624 Pécs, Hungary; 8 Department of Pharmacology and Toxicology, University of Veterinary Medicine, István Utca 2, H-1078 Budapest, Hungary; 9 Department of Laboratory Medicine, Medical School, University of Pécs, H-7624 Pécs, Hungary; 10 Molecular Medicine Research Group, Szentágothai Research Centre, University of Pécs, H-7624 Pécs, Hungary

**Keywords:** phylogenetic analysis, recombination analysis, aviadenoviruses, poultry, inclusion body hepatitis, *Aviadenovirus gallinae*

## Abstract

Fowl adenovirus D is the main cause of inclusion body hepatitis in chickens. Whole genome sequencing was carried out to enrich the genomic sequence database using field isolates of FAdV-D. Out of 44 newly determined genomes, 43 were classified into FAdV-2/-11 and 2 into FAdV-3; no FAdV-9 was identified. Whole-genome based phylogeny showed that FAdV-3 was more distantly related to FAdV 9 and FAdV-2/-11 than FAdV-9 and FAdV-2/-11 to each other. Whole-genome sequence homology analysis revealed that the full-length FAdV-3 genome harbored a ~12 kbp fragment of the genome that shared moderate sequence homology with representative strains of other FAdV-D serotypes but high relatedness with only the FAdV-3 strain whose full-genome is available in GenBank. A closer look onto the fiber and the penton genes of our FAdV-3 isolate identified putative recombination events; both the fiber and the penton coding genes shared fragments originating from FAdV-9. Of interest, ORF19 displayed a close relationship with the homologous genomic region of some FAdV-E strains (amino acid sequence homology, up to 82%). Thus, although FAdV-3 is classified into FAdV-D, the genomic structure of FAdV-3 appears to result from multiple heterotypic and heterologous recombination events. This study highlights the unique origin of FAdV-3.

## Simple Summary

Adenoviral inclusion body hepatitis in chicken has been, in part, associated with serotypes of fowl adenovirus D. We determined the genome of 44 FAdV-D isolates collected from parts of the world, including 43 FAdV-2/-11 and a rare FAdV-3. This study focused on the sole FAdV-3 isolate, showing evidence of a mosaic structure of the viral genome. These findings highlight the need for full-genome analysis of rarely isolated virus variants to better reconstruct their evolution and epidemiology.

## 1. Introduction

All the evidence suggests that birds are the sole hosts of the genus *Aviadenovirus* [1]. The aviadenovirus particle is similar in shape and size to other adenoviruses, although some members of the genus carry two fibers at each apex of the icosahedron. The aviadenovirus genome is among the largest within the *Adenoviridae*, measuring approximately 38.6 to 45.8 kbp. The genomic organization of aviadenoviruses differs from that of other adenoviruses. The genus *Aviadenovirus* contains at least 21 species [1]. Aviadenoviruses of chicken, fowl adenoviruses (FAdVs), are classified into five species: *Aviadenovirus ventriculi* (fowl adenovirus A; FAdV-A), *Aviadenovirus quintum* (FAdV-B), *Aviadenovirus hydropericardii* (FAdV-C), *Aviadenovirus gallinae* (FAdV-D), and *Aviadenovirus hepatitidis* (FAdV-E). This classification is based primarily on the genomic organization and the phylogenetic relationship among strains [1]. FAdVs are globally prevalent, and commercial flocks of chickens can be heavily affected by FAdV infections. In chickens, the main FAdV-associated pathologies are gizzard erosions (GEs), hepatitis-hydropericardium syndrome (HHS) and inclusion body hepatitis (IBH) [2]. Field reports on the etiologic link have shown that members of FAdV-C and FAdV-A are mainly isolated from HHS and GE outbreaks, respectively, whereas FAdV-D and FAdV-E are most often isolated from IBH cases. However, the clinical spectrum is more complex, as many FAdV infections are subclinical, while others show non-specific manifestations. In the latter cases, FAdVs are usually identified as secondary pathogens whose contribution to the observed morbidities and mortalities is not fully understood [3].

FAdV-D comprises four serotypes, FAdV-2, -3, -9 and -11. Early reports have distinguished between the FAdV-2 and FAdV-11 serotypes based on serological data; however, more recent studies have been unable to determine the genetic basis for this separation using sequence data. FAdV-D infections have been reported from numerous countries worldwide, mainly from IBH outbreaks. For example, FAdV-2/-11 and FAdV-3 have been reported from Hungary, FAdV-2 from Japan, FAdV-3 and FAdV-11 from Korea, FAdV-11 from Australia, FAdV-2 from South Africa, FAdV-2 and 3 from Italy, FAdV-11 from Brazil and FAdV-2/-11 from China. Overall, FAdV-D serotypes 2 and 11 represent emerging strains in parts of the world, while serotypes 3 and 9 appear to be less prevalent. Furthermore, FAdV -2/-11 and -3 have been detected in wild birds, suggesting that avian species that belong to Anseriformes, Strigiformes, and Columbiformes may serve as reservoirs of these aviadenovirus serotypes or their very close relatives [4,5,6,7,8,9,10,11,12,13].

In a recent paper, we reported the wide geographic distribution of pathogenic FAdVs based on the results of a multi-year diagnostic study [3]. In that study, we used complex laboratory testing that included the sequencing and phylogenetic analysis of the viral DNA polymerase. This preliminary genetic characterization was suitable for the identification of FAdV species but was less informative from an epidemiologic and evolutionary viewpoint [3]. In the present study, we extended the number of full genomes of FAdV-D isolates and analyzed a rare variant, FAdV-3, in more detail. Additionally, the data generated will be useful to better understand the clinical role of serotypes and individual genome variants circulating in the field, and the data can be used in descriptive epidemiologic surveillance as well as in phylodynamic and phylogeographic analyses.

## 2. Materials and Methods

### 2.1. Virus Strains

FAdV-D isolates in this study represented a large strain collection comprising 125 partially characterized strains [3]. The primary identification of these 125 FAdV-D strains was based on a combination of laboratory techniques, such as virus isolation in embryonated eggs and cell cultures, and molecular detection techniques, such as conventional and real-time PCR, restriction fragment length polymorphism and/or the nucleotide sequencing of the amplified gene. All diagnostic procedures were carried out at the Scientific Support and Investigation Unit, Ceva-Phylaxia Co. Ltd., Budapest, Hungary, over the period spanning 2008 and 2019. Further details are shared in the recent publication by Kiss and coworkers [3].

### 2.2. Genome Sequencing

All FAdV-D isolates were kept at −70 °C until DNA extraction and genome sequencing. In brief, the consensus genome sequences were obtained by using next-generation sequencing. The adenoviral DNA was extracted from cell culture supernatants using the ZiXpress-32 Viral Nucleic Acid Extraction Kit and ZiXpress-32 Automated Nucleic Acid Purification Instrument (Zinexts Life Science Corp., New Taipei City, Taiwan). Libraries were prepared using the Illumina Nextera XT DNA Library Preparation Kit (Illumina, San Diego, CA, USA) and published protocols [14,15]. Sequencing was carried out on Illumina 500 equipment. Single-end reads of 150 nucleotides were generated.

### 2.3. Sequence Analysis

Genome assemblies were carried out by using the Geneious Prime^®^ 2022.2.2 software (Biomatters Ltd., Auckland, New Zealand). The coding potential was predicted based on reference genomes with the same software package. Sequence similarities were compared to GenBank records by Basic Local Alignment Search Tool [16]. Multiple alignments of the individual genes as well as the complete genomes were prepared in the MAFFT aligner [17]. Gene-wise and whole genome-based phylogenies were performed with the maximum-likelihood method and optimized substitution models (whole genome, TPM2 + F + R8; DNA polymerase, HKY + F + R2; penton, HKY + F + I; fiber, TPM2 + F + R3; hexon, TN93 + R4 model) in MEGA 11 (version 11.0.13.) and IQ-TREE [18,19]. Bootstrap support values for the maximum likelihood trees were calculated using 1000 iterations. Genome-wide homology was assessed by sliding window analysis (window size, 200 bp; step, 20 bp; model, Jukes-Cantor) with the SimPlot++ tool [20].

### 2.4. Data Reposition

The assembled FAdV-D genome sequences were deposited in GenBank under the accession numbers PP471920 to PP471963. Additional information on FAdV-D isolates can be found in Table 1 and the Supplementary Materials.

## 3. Results and Discussion

The FAdV-D isolates available for this study were collected from 18 countries and four continents (Table 1, Supplementary Materials). The 45 isolates initially selected represented 36% of all FAdV-D strains isolated from clinical and pathological samples between 2008 and 2019. The viral genome was successfully assembled for 44 out of 45 FAdV-D isolates; the genome of 1 isolate could not be fully determined due to the low number of specific sequence reads. Genome sequences were assembled with a sequence depth ranging from 13X to 3873X (median, 1344X). The length of consensus genomes varied between 43,219 bp and 45,138 bp (Supplementary Materials). Each viral genome was predicted to encode 36 ORFs. Nucleotide sequences were used as queries to determine the type specificity. As a result of a genome similarity search with the Blast engine and based on preliminary phylogenetic analyses, the study strains were grouped into two clusters. The larger branch contained 43 strains most closely related to the combined FAdV-2/-11 cluster; the detection of these strains spanned the entire collection period and the entire study area. A single isolate was typed as FAdV-3; this strain was detected in France in 2009. The fourth serotype of FAdV-D, FAdV-9, was not identified among the selected isolates. To gain insight into the relationship among globally circulating FAdV-D strains, the study strains (n = 44) were analyzed with several reference strains (n = 31), whose full genome was deposited in GenBank. The genome sequences and selected genes were analyzed phylogenetically; moreover, a gene- and genome-wide nucleotide sequence similarity search was performed by using sliding window analysis.

**Figure 1:**
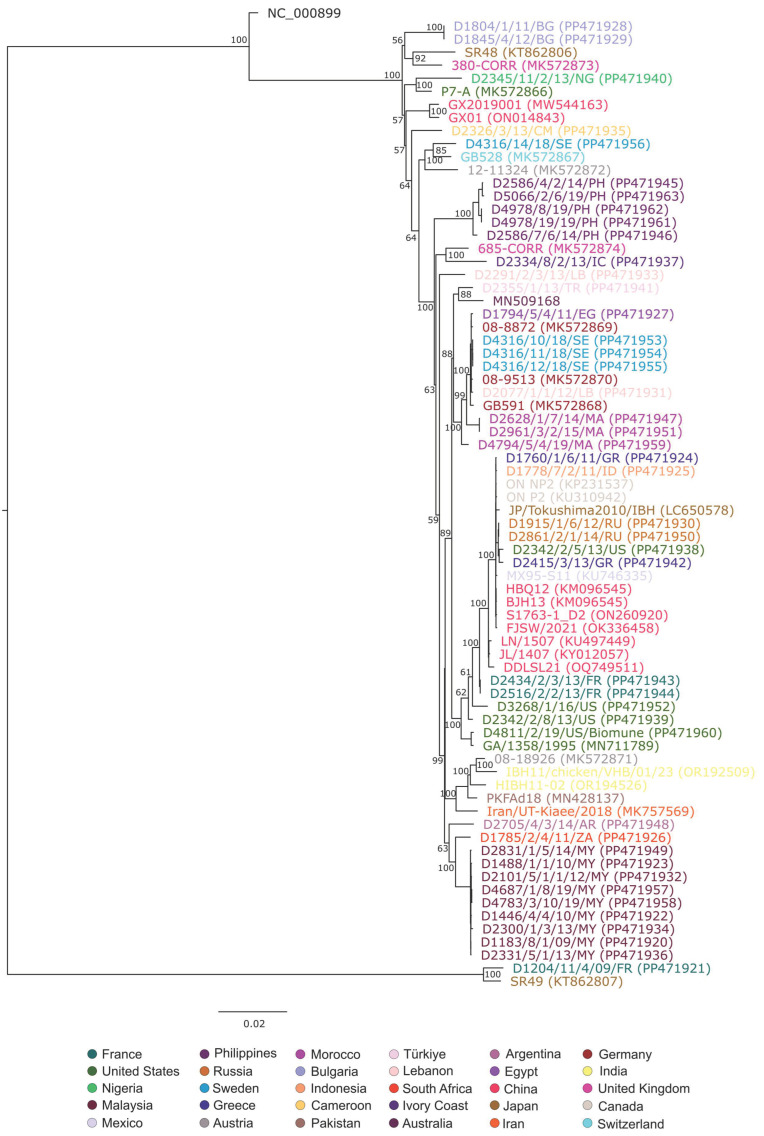
Nucleotide sequence-based phylogenetic analysis of FAdV-D strains using the whole genome sequence (color codes of taxa; identical colors indicate shared geographical origin).

Phylogenetic analysis using whole-genome sequences was performed after the deletion of large unequally aligned genomic regions (spanning the region from nt 37,829 to 39,278 in strain SR48 [KT862806]), which resulted in significant sequence length differences among the genomes. Whole-genome based phylogeny (Figure 1) showed that all FAdV-2/-11 strains formed a large cluster containing numerous strains (sequence identity, ≥97.4%) and FAdV-9 clustered with this large branch (sequence identity range, 95.5% to 96.6%). A small cluster contained the two known FAdV-3 strains (SR49 and D1204/11/4/09/FR) with available full-genome sequences, and this cluster was more distantly related to all other FAdV-D genomes (sequence identity ranges, with the FAdV-2/-11 cluster, 89.5% to 90.5%; with FAdV-9, 91.9%).

**Figure 2:**
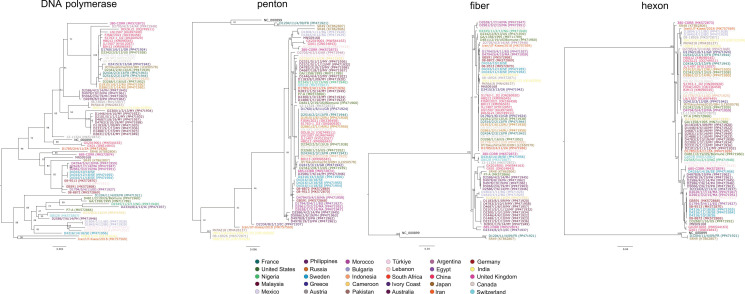
Nucleotide sequence based phylogenetic analysis of FAdV-D strains using the DNA polymerase, the penton, the fiber and the hexon genes (color codes of taxa; identical colors indicate shared geographical origin).

Additional phylogenetic analyses were carried out using the nucleotide alignments of the DNA polymerase, the hexon, the penton and the fiber coding genes (Figure 2). These analyses revealed that DNA polymerase forms two statistically significant clusters; of interest, both branches contained a large number of FAdV-2/-11 strains. The sole FAdV-9 strain clustered with the larger group containing somewhat more taxa, whereas the two FAdV-3 strains showed a different branching pattern. In particular, the GenBank reference FAdV-3 strain, SR49, was genetically more closely related to the FAdV-9 strain than the other FAdV-3 strain sequenced in this study. The three main virion components expressed on the surface (i.e., the hexon, the fiber and the penton) showed some differences in the tree topologies. In particular, the FAdV-2/-11 cluster formed a common branch in the hexon and the fiber gene phylogenies and also formed a statistically supported branch in the penton tree. The position of FAdV-3 and FAdV-9 strains was slightly different. In the hexon tree, FAdV-3 and FAdV-9 formed a common cluster and the genetic distance between FAdV-3 and FAdV-9 was similar to that observed within the FAdV-2/-11 cluster (97.6–100% vs. 96.8–100%). In the fiber gene tree, although forming a common cluster, the sole FAdV-9 strain appeared to be more closely related to strains in the FAdV-2/-11 cluster than the FAdV-3. A similar pattern was seen in the penton tree; in addition, the FAdV-3 strain from France appeared to be in an intermingled position between the FAdV-9 and the reference FAdV-3 strain.

**Figure 3:**
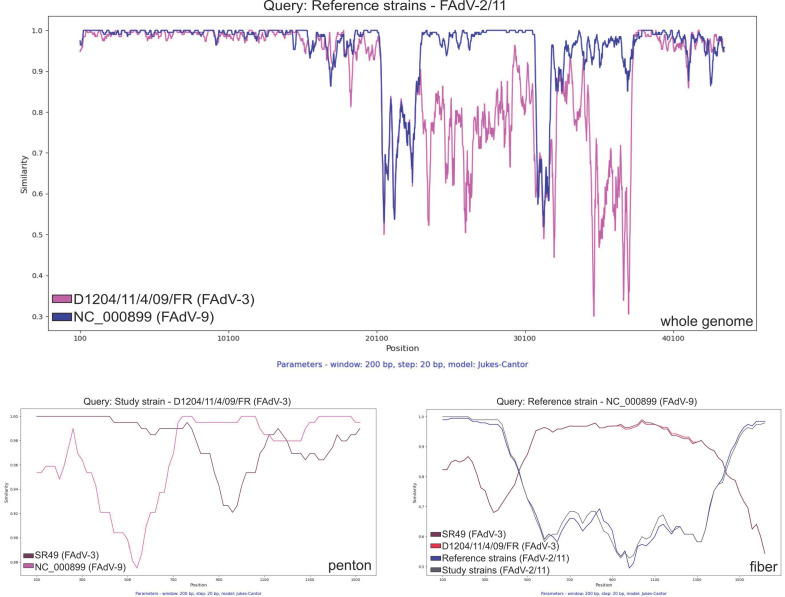
Simplot analyses show the sequence similarities along the whole genome (upper panel) and some genes (i.e., penton, fiber; lower panels) among the selected strains.

This peculiar pattern of gene-wise phylogenies prompted us to further investigate the inter-serotype sequence relationships, and we performed gene- and genome-wide sequence similarity analysis that permitted the identification of conserved and diversified genomic regions (Figure 3). Consistent with the report by Schachner and co-workers [21], a ~12 kbp segment spanning the hexon coding region to the end of the fiber coding region was uniquely diversified in the FAdV-3 strains, although as delineated in the phylogenetic analysis section, both the hexon- and the fiber coding regions in this diversified segment of FAdV-3 shared greater similarity with FAdV-9. However, when analyzing the fiber coding gene by SimPlot, the FAdV-9 strain shared greater similarity to both historical and more recent FAdV-2/-11 strains at the 5’ and 3’ ends and greater similarity to FAdV-3 strains in the central region of the gene, which is consistent with past recombination events in the fiber coding gene. Of interest, approximately half of the penton coding gene of the FAdV-3 strain from France shared similarity with the reference FAdV-3 strain, while the other half of the gene was more similar to the reference FAdV-9 strain, a finding that suggests an inter-serotype recombination event independent from the event observed in the fiber coding gene (Figure 1). Another genomic region, ORF19, coding for a lipase-like protein is very divergent and is often the subject of genetic analyses; these analyses have shown that recombination may occur in this region even between distantly related adenoviruses [11,22,23,24]. When analyzing the 723 amino acid long-predicted protein of ORF19, both FAdV-3 strains shared moderate genetic relatedness with FAdV-9 and FAdV-2/-11 strains (up to 65% identity) and greater sequence relatedness (up to 82% identity) with selected FAdV-E strains (e.g., 13-19395, 09-8330).

These data together suggest that the genome of FAdV-D serotypes, including the sparsely sequenced FAdV-3, evolved through multiple, consecutive recombination events involving both heterotypic FAdV-D serotypes and aviadenoviruses with either known serotypes (within FAdV-E) or unknown serotypes.

## 4. Conclusions

Our analyses confirmed the notion from epidemiologic surveillance conducted in parts of the world that the FAdV-2/-11 cluster represents a globally prevalent FAdV-D serotype; the vast majority of FAdV-D isolates in our strain collection (~98%) carried this type specificity. In addition, we confirmed the conserved nature of genomic organization within serotypes of FAdV-D. In general, the genome of FAdV-2/-11 strains was more similar to FAdV-9, while FAdV-3 had a long stretch in the central region of the genome that may have evolved from an ancient recombination event. Ancestral recombination events within and between homologous FAdV-D strains that involved genome fragments of various lengths may have been followed by more recent inter-serotype recombination events that further shaped the viral genome structure. The putative FAdV-3/FAdV-9 inter-serotype recombination event detected in the gene coding for the penton antigen appears to represent a more recent evolutionary leap. The question of whether similar major evolutionary leaps could lead to changes in the antigenic features of circulating FAdV-D strains could be the subject of future studies. The genome sequence data reported here may contribute to a better understanding of the evolutionary mechanisms, epidemiological dynamics and phylogeography of emerging FAdV-D strains and may help devise future control and prevention measures against FAdV-D-associated IBH outbreaks.

## Figures and Tables

**Table 1:** Information on FAdV-D strains sequenced in this study.

Strain ID	Year of Isolation	Country of Origin	Isolated from Sample	Production Type/Age	Sero-/Genotype
D1183/8/1/09/MY	2009	Malaysia	bursa	broiler/D45	FAdV-2/FAdV-11
D1204/11/4/09/FR	2009	France	liver	slow-grown broiler/D56	FAdV-3
D1446/4/4/10/MY	2010	Malaysia	cecal tonsil	broiler/D34	FAdV-2/FAdV-11
D1488/1/1/10/MY	2010	Malaysia	liver	broiler/D20	FAdV-2/FAdV-11
D1760/1/6/11/GR	2011	Greece	liver	broiler	FAdV-2/FAdV-11
D1778/7/2/11/ID	2011	Indonesia	proventriculus	broiler/D20	FAdV-2/FAdV-11
D1785/2/4/11/ZA	2011	South Africa	liver	broiler embryo	FAdV-2/FAdV-11
D1794/5/4/11/EG	2011	Egypt	cecal tonsil	broiler/D39	FAdV-2/FAdV-11
D1804/1/11/BG	2011	Bulgaria	liver	broiler/D10	FAdV-2/FAdV-11
D1845/4/12/BG	2012	Bulgaria	liver	broiler/D10	FAdV-2/FAdV-11
D1915/1/6/12/RU	2012	Russia	liver	broiler/D19	FAdV-2/FAdV-11
D2101/5/1/1/12/MY	2012	Malaysia	cecal tonsil	broiler/D42	FAdV-2/FAdV-11
D2291/2/3/13/LB	2013	Lebanon	cecal tonsil	broiler/D42	FAdV-2/FAdV-11
D2300/1/3/13/MY	2013	Malaysia	cecal tonsil	broiler/D36	FAdV-2/FAdV-11
D2326/3/13/CM	2013	Cameroon	intestine	broiler/D42	FAdV-2/FAdV-11
D2331/5/1/13/MY	2013	Malaysia	liver	broiler/D35	FAdV-2/FAdV-11
D2334/8/2/13/IC	2013	Ivory Coast	cecal tonsil	broiler/D42	FAdV-2/FAdV-11
D2342/2/5/13/US	2013	United States	liver	broiler	FAdV-2/FAdV-11
D2342/2/8/13/US	2013	United States	liver	broiler	FAdV-2/FAdV-11
D2345/11/2/13/NG	2013	Nigeria	cecal tonsil	broiler/D32	FAdV-2/FAdV-11
D2355/1/13/TR	2013	Türkiye	cecal tonsil	broiler/D40	FAdV-2/FAdV-11
D2434/2/3/13/FR	2013	France	cecal tonsil	broiler/D29	FAdV-2/FAdV-11
D2516/2/2/13/FR	2013	France	liver	broiler/D26	FAdV-2/FAdV-11
D2586/4/2/14/PH	2014	Philippines	cecal tonsil	broiler/D35	FAdV-2/FAdV-11
D2586/7/6/14/PH	2014	Philippines	cecal tonsil	broiler/D29	FAdV-2/FAdV-11
D2628/1/7/14/MA	2014	Morocco	cecal tonsil	broiler/D36	FAdV-2/FAdV-11
D2705/4/3/14/AR	2014	Argentina	proventriculus	broiler/D38	FAdV-2/FAdV-11
D2831/1/5/14/MY	2014	Malaysia	cecal tonsil	broiler/D38	FAdV-2/FAdV-11
D2861/2/1/14/RU	2014	Russia	gizzard	broiler/D20	FAdV-2/FAdV-11
D2961/3/2/15/MA	2015	Morocco	kidney	broiler/D36	FAdV-2/FAdV-11
D3268/1/16/US	2016	United States	liver	broiler/D7	FAdV-2/FAdV-11
D4316/10/18/SE	2018	Sweden	cecal tonsil	n. a.	FAdV-2/FAdV-11
D4316/11/18/SE	2018	Sweden	liver	n. a.	FAdV-2/FAdV-11
D4316/12/18/SE	2018	Sweden	liver	n. a.	FAdV-2/FAdV-11
D4316/14/18/SE	2018	Sweden	liver	n. a.	FAdV-2/FAdV-11
D4687/1/8/19/MY	2019	Malaysia	cecal tonsil	broiler/D38	FAdV-2/FAdV-11
D4783/3/10/19/MY	2019	Malaysia	cecal tonsil	broiler/D37	FAdV-2/FAdV-11
D4794/5/4/19/MA	2019	Morocco	liver	broiler/D40	FAdV-2/FAdV-11
D2077/1/1/12/LB	2012	Lebanon	cecal tonsil	broiler/D39	FAdV-2/FAdV-11
D2415/3/13/GR	2013	Greece	cecal tonsil	broiler/D45	FAdV-2/FAdV-11
D4811/2/19/US	2019	United States	liver	n. a.	FAdV-2/FAdV-11
D4978/19/19/PH	2019	Philippines	liver	broiler/D32	FAdV-2/FAdV-11
D4978/8/19/PH	2019	Philippines	liver	broiler/D32	FAdV-2/FAdV-11
D5066/2/6/19/PH	2019	Philippines	bursa	broiler/D21	FAdV-2/FAdV-11

n. a.: not available.

## Data Availability

The dataset generated for this study can be found in GenBank (see Section 2.4 and Supplementary Materials).

## Supplementary Materials

The following supporting information can be downloaded at: https://www.mdpi.com/article/10.3390/ani15040508/s1, Table S1: Background information on FAdV-D strains sequenced in this study.
